# A Conceptual Model of Refugee Family Dynamics: A Study with Sri Lankan Tamils

**DOI:** 10.3390/ijerph22020169

**Published:** 2025-01-26

**Authors:** Miriam Kuttikat, Marianne B. Lund, David Chan, Indranil Sahoo

**Affiliations:** 1 School of Social Work, Virginia Commonwealth University, Richmond, VA 23284, USA; kuttikatm@vcu.edu (M.K.); lundm@vcu.edu (M.B.L.); 2 Department of Mathematics and Applied Mathematics, Virginia Commonwealth University, Richmond, VA 23284, USA; dmchan@vcu.edu; 3 Department of Statistical Sciences and Operations Research, Virginia Commonwealth University, Richmond, VA 23284, USA

**Keywords:** migration Stressors, repatriation, migrant family dynamics, migrant health intervention

## Abstract

Sri Lankan Tamil refugees have endured over four decades of protracted displacement in southern India. This paper synthesizes findings from four studies conducted between 2005 and 2018 among refugees residing in the Gummidipoondi and Trichy refugee camps in the state of Tamil Nadu, India. Framed by a Community Based Participatory Research, in collaboration with the Organization for Eelam Refugee Rehabilitation (OfERR) and Community Advisory Board, these studies aimed to investigate the challenges related to migration stressors, mental health, family dynamics, and resource utilization of the Sri Lankan refugees living in India. The association of Psychological Distress and Migration Stress (PDMS) study examined migration stressors influencing psychological distress among refugees. The intergenerational Conflict and Community Readiness (ICCR study) assessed community readiness for repatriation and intergenerational conflict employing semi-structured qualitative interviews. The Family Dynamics (FD) mixed-method study investigated longitudinal associations between migration stressors, resource utilization, family dynamics, and health outcomes among Sri Lankan Tamil refugees. The Parenting Processes and Intervention Development (PPID) Study incorporated perspectives of community health workers through qualitative exploratory research. Integrating these findings, the research developed (1) Conceptual Model of Refugee Family Dynamics and (2) Framework for Refugee Health Intervention. These models provide a culturally sensitive framework for future interventions to improve family well-being among refugee populations.

## 1. Introduction

For more than four decades, Sri Lankan Tamil refugees have experienced protracted displacement, primarily in the southern Indian state of Tamil Nadu. This community of displaced individuals, which fled the civil war between the Sri Lankan government and the Liberation Tigers of Tamil Eelam (LTTE) from 1983 to 2009, constitutes one of the most enduring refugee crises in South Asia. Over 300,000 refugees have endured statelessness, acculturative stress, and a lack of formal resettlement pathways [1,2]. With nearly 125,000 refugees living in over 100 camps across Tamil Nadu, this population epitomizes the challenges posed by prolonged displacement and systemic marginalization [3].

The forced migration of Tamils from Sri Lanka occurred in several waves, starting in the early 1980s and intensifying with subsequent phases of violence during the civil war [4]. This period of conflict, rooted in ethnic tensions between the Sinhalese majority and Tamil minority and culminating in the LTTE’s defeat in 2009 after a 26-year civil war, caused widespread destruction, civilian casualties, and mass displacement [5]. These waves of migration, coupled with unresolved political tensions, have resulted in a protracted refugee situation that continues to this day [5,6,7]. Prolonged displacement has contributed to a range of transmigration stressors, including loss of resources, limited access to legal and social services, and pervasive uncertainty regarding resettlement or repatriation [8,9,10]. These cumulative stressors have had profound psychosocial impacts on Tamil refugee families, affecting intergenerational family dynamics, mental health, and overall well-being [11,12,13].

Historically, forced migration research has been conducted through two primary and sometimes divergent perspectives. One approach centers on a trauma-focused lens, emphasizing the acute psychological consequences of conflict and displacement, whereas the other prioritizes psychosocial factors influencing mental health outcomes [8]. Over the past decade and a half, ecological models have emerged to address the multifaceted nature of forced migration experiences, acknowledging the interplay of individual, relational, and societal factors that shape the mental health trajectories of refugee populations [12,14,15]. Despite this growing recognition of complexity, a notable gap persists in the literature with respect to examining trauma, psychosocial stressors, resource utilization, and the ways in which these factors collectively influence family dynamics. This paper aims to address this gap by focusing on family relationships within the Sri Lankan Tamil refugee population in South Asia. In doing so, it contributes to the broader field of refugee studies by offering findings that illuminate how a confluence of pre- and post-migration stressors intersect to affect familial cohesion, adaptation, and well-being.

## 2. Background

### Study Setting

India has the highest number of Sri Lankan Tamils refugees outside of Sri Lanka. Today over 125,000 of these Sri Lankan Tamil refugees live in India, with 90,000 living in refugee camps [16]. During the different migration waves in 1984, 1999 and 2006, approximately 20,000 Sri Lankan Tamil refugees came to Southern India every year [17]. Most of the refugee camps, 115 of the 123, are in the Southern Indian State of Tamil Nadu due to the linguistic and ethnic kinship that exists between Sri Lankan Tamils and the native residents [18]. The research for all four studies discussed here was conducted by Dr. Kuttikat (First Author and Principal Investigator, (PI)) at the Gummidipoondi and Trichy refugee camps in Tamil Nadu, India, which houses approximately 2500 families of Sri Lankan Tamil refugees displaced by civil unrest. The PI has been involved in research at this site since 2005, fostering a long-term relationship with the community. This ongoing collaboration has been key to building trust and ensuring the study’s success. All study procedures were approved by the PI’s institutional review board.

Over the past 13 years, the PI and colleagues have conducted extensive research within Indian refugee camps to examine complex migration stressors, family dynamics and psychological well being. Grounded in Community-Based Participatory Research (CBPR) principles, the work has prioritized recognizing community identity, leveraging local strengths, fostering equitable collaboration, and integrating knowledge with action to benefit all stakeholders [19,20]. A Community-Based Advisory Board (CAB), composed of camp authorities, Tamil refugee elders, camp residents, the organization ELAM Refugees (OfERR), the research team, and funding agencies ensured the operationalization of CBPR principles within cultural norms. The primary participants in the CAB were the Organization for Eelam Refugee Rehabilitation (OfERR) and the Community Elders. OfERR, established in 1984 by Sri Lankan Tamil refugees themselves for coordinating support for the Tamil refugees living in the Indian camps, served as the community collaborator. The research studies also benefited from sustained engagement with the CAB, which actively supported the project for 13 years (2005–2018) until its completion. The CBPR framework ensured that the research remained grounded in the needs and perspectives of the refugee community, with active involvement from the CAB in guiding the process.

The PI utilized Rowson’s FAIR (Fairness, Autonomy, Integrity, and Results) framework [21] as an ethical guide to develop an adaptive research plan that would prepare the PI for the uncertain circumstances that often characterize international refugee research. Rowson’s FAIR framework specifically helped the PI to integrate the concepts of fairness, autonomy, integrity, and results into her interdisciplinary (social work, community development, and mental health) international research by negotiating many cross-cultural socio-political dilemmas with the Sri Lankan Tamil refugee population in India. The CAB played a critical role in developing culturally appropriate research design, research questions, translation of research documents, oral and written consent procedures and data collection methods and fostering community trust. An additional contribution of the CAB was to train local community health workers to lead the data collection process, ensuring that procedures were both respectful and relevant to participants.

## 3. Methods

**Figure 1:**
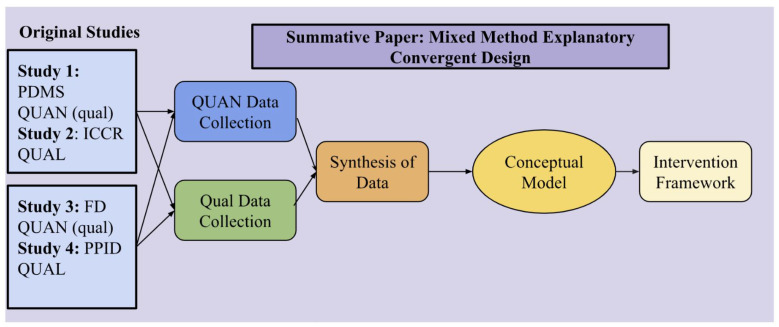
Mixed-Methods Explanatory Convergent Design: Pathway to Conceptual Model and Intervention Framework.

This summative paper integrates findings from four studies conducted over a thirteen-year period (2005–2018), each examining aspects of migration stressors, family dynamics, and psychological well-being among Sri Lankan Tamil refugees in India. The synthesis of data presented here follows a mixed-methods explanatory convergent design [22,23], illustrated in Figure 1. In this design, quantitative and qualitative data are collected and analyzed independently, and then converged to generate integrated insights. The explanatory emphasis places quantitative data at the forefront (QUAN), with qualitative data providing depth and context to enrich the statistical findings.

A key feature of this approach was the point of data convergence, which occurred only after each quantitative and qualitative strand was fully analyzed in studies 1–4 [24,25]. Quantitative data was gathered using standardized measures, survey instruments, and demographic variables focused on migration stressors, family functioning and mental health outcomes, while qualitative data comprised in-depth interviews, focus group discussions, and participatory community dialogues. Collecting and analyzing data in this manner enabled a multi-layered exploration of how migration stressors impact refugee well-being, revealing both generalizable patterns and nuanced cultural dynamics.

Reliability was bolstered by consistent data collection protocols across studies, including psychometrically valid instruments and rigorously maintained field procedures. Validity was strengthened through the triangulation of quantitative and qualitative findings, supplemented by iterative discussions with CAB. The CAB, composed of camp authorities, refugee elders, local organizations, and research team members, served as a cultural and ethical checkpoint throughout the research. Its involvement ensured that methodological choices remained aligned with the community’s values and experiences. This process also fostered relationships of trust and transparency, essential for conducting longitudinal research in a refugee context.

**Figure 2:**
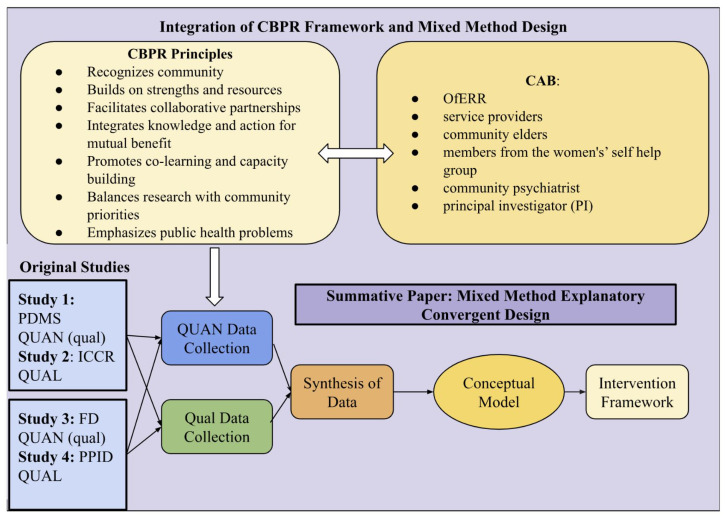
Integration of CBPR Framework and Mixed Method Design.

The purpose of this summative paper is to synthesize the collective findings in order to generate an ecologically informed conceptual model of refugee family dynamics and inform a framework for future interventions. Figure 2 demonstrates how the CBPR framework [19,20] was overlaid onto the mixed-methods process, illustrating how principles of collaboration, shared decision-making, and local relevance were embedded at every stage. This synergy of the explanatory convergent design with CBPR principles enhanced both methodological rigor and cultural responsiveness.

This summative paper maps the impact of refugee experiences on family dynamics. Family dynamics, from a social-ecological perspective, refers to the ways in which family members relate to one another in their current living environment [8,13,15,26]. From the PI’s work and existing literature on Sri Lankan Tamil refugee families [13,27], it is clear that exposure to political violence and subsequent stressful life events disrupt family dynamics such as parenting style, communication and responsiveness, which in turn contribute to deterioration of health. To understand the scope of these complexities, the PI approached such problems with a framework that looks beyond individuals—i.e., one that is social and ecological [26]. Socio-ecological models align with the conceptual framework emphasizing the role of refugee family dynamics on the interaction between refugees and their living environments. Our research contributes to the existing literature examining how both trauma and ecological factors impact pre- and post- migration mental health, with a specific focus on the impact on intergenerational family relationships.

### 3.1. Study 1: Association of Psychological Distress and Migration Stressors

The association of Psychological Distress and Migration Stress (PDMS) study (2005–2009) examined the impact of migration stressors on Sri Lankan Tamil refugees psychological well being, focusing on four key topics. First, it explored how demographic characteristics and migration stressors influence refugees’ psychological distress, especially refugees’ with families and children [28]. Second, it investigated the effects of pre- and post-migration trauma on mental health, offering insights for trauma-informed social work interventions [29], with special consideration given to international refugee research, emphasizing the importance of culturally sensitive engagement with vulnerable populations [30]. Third, the study included an analysis of the socio-political as well as economic barriers preventing successful repatriation. Finally, the readiness for repatriation was contrasted to the desire to remain, and the impact of these intentions on intergenerational family dynamics was discussed. The synthesis of this original work contributed to a detailed conceptual policy framework proposed as a guide for future reintegration efforts [18].

**Figure 3:**
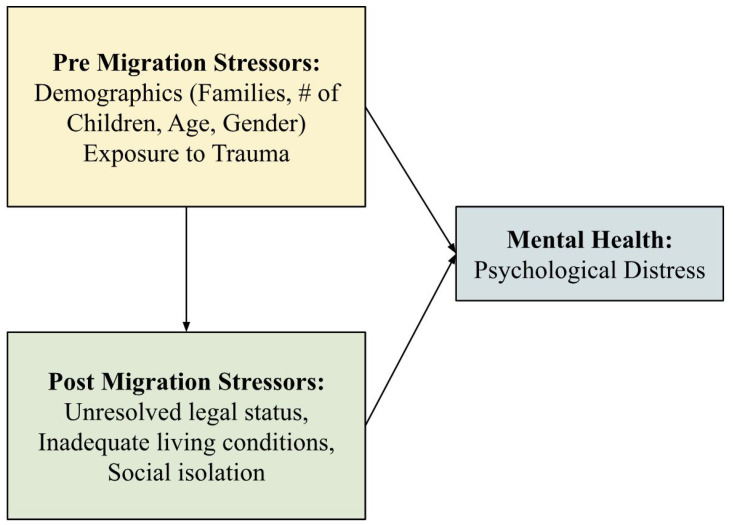
The impact of pre- and post-migration stressors on mental health.

The PDMS study aimed to examine the relationship between migration stressors and psychological distress, as illustrated in Figure 3. Quantitative assessments focused on individual and family-level stressors on refugee mental health. Qualitative data was collected to provide deeper contextual insights into the quantitative results. Institutional Review Board (IRB) approval was obtained from the University of Toronto (IRB #23087; 2 October 2008), and the study was funded by the University of Toronto Fellowship Social Science, Humanities Research Council Seed Grant, and the Canadian Health Research Fellowship. Fifty participants were recruited through purposive sampling from various subdivisions of the camp. Participants had an average age of 34.9 years, with a male majority (60.2%). Almost half had some secondary education (49.4%), were married (42.2%), and had children (43.4%). The Harvard Trauma Questionnaire (HTQ), Post-Migration Living Difficulties Questionnaire (PMLDQ), and Symptoms Checklist-90-R (SCL-90-R) were employed to assess pre-migration stressors, post-migration stressors, and psychological distress, respectively [28].

The PDMS study found that both pre- and post-migration stressors were significant predictors of psychological distress (see Figure 3). Each unit increase in daily migration stressors was associated with a 1.16-unit increase in psychological distress (p<0.001). Family dynamics also played a role, with higher distress levels among participants who were married (p=0.036), had children (p=0.021), or lived with their families (p<0.001) [29]. Further analysis supported these findings, revealing that refugees in India faced heightened post-migration stressors due to unresolved legal status, inadequate living conditions, and ongoing social isolation [11,31].

**Figure 4:**
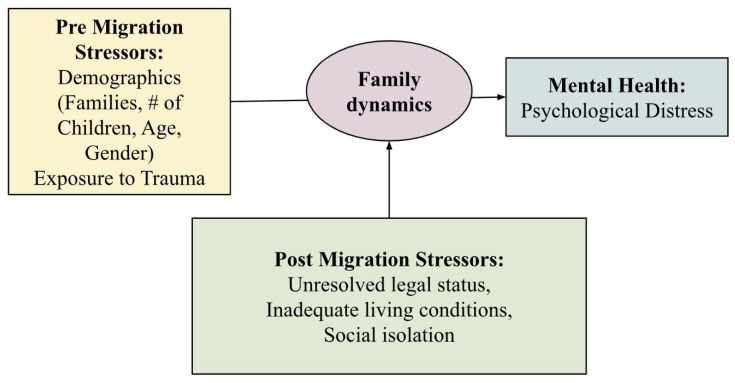
Family dynamics as a moderating role on mental health.

Family dynamics emerged as a significant moderating factor of mental health for both pre- and post-migration stress, as illustrated in Figure 4. The study revealed that family dynamics further contributed to psychological outcomes, particularly in relation to family size. Ref. [28] identified a positive correlation between the number of children and levels of psychological distress. Larger families faced increased economic burdens, amplified by limited camp resources and social isolation, conditions found to be more pronounced in India than other diaspora settlements [3]. These findings emphasize how family-related stressors exacerbate mental health challenges for refugees, aligning with the core conclusions of the PDMS Study.

The qualitative component of the PDMS study provided deeper insights into how migration stressors, including poor repatriation policies and insufficient support, exacerbate refugee suffering. Many participants expressed uncertainty regarding repatriation intensified their daily psychological distress. Anecdotal evidence highlighted intergenerational conflicts within refugee families, adding complexity to family dynamics and pointing to the need for interventions that address both individual trauma and family cohesion [28].

In summary, the findings from this PDMS study highlight the multiple layers of challenges faced by Sri Lankan Tamil refugees. Both pre- and post-migration stressors, family dynamics, and uncertainty regarding repatriation contribute to prolonged psychological distress. Addressing these issues requires interventions that not only focus on individual mental health but also address systemic challenges, support for family cohesion, and the offer of sustainable solutions for repatriation [28,29].

### 3.2. Study 2: Intergenerational Conflict and Community Readiness for Repatriation of Sri Lankan Tamil Refugees in India

This qualitative study (2013–2014) aimed to explore the intergenerational Conflict and Community Readiness (ICCR) for repatriation of Tamil refugees to their country of origin, which is Sri Lanka, focusing on their support systems, concerns, resources, and strategies for returning to their homeland. intergenerational conflict in refugee families often stems from asynchronous adaptation, where younger generations assimilate into the host culture faster than their elders. This leads to tensions as values, expectations, behaviors, and communication styles diverge [29,32,33]. Factors such as differences in language acquisition, access to education and employment, and pressures to either conform or preserve cultural identity contribute to this conflict. These tensions are further intensified by the psychological stress and trauma of displacement, which result in differing coping mechanisms, expectations, and goals between generations [1,33,34].

The PI conducted semi-structured interviews to assess the community’s readiness for repatriation, focusing on six main areas: awareness of repatriation, community support, concerns, leadership, available resources, and strategies for overcoming challenges. The study was approved by the Virginia Commonwealth University Institutional Review Board (IRB #HM15259; 24 April 2013), and was funded by Virginia Commonwealth University School of Social Work Internal Funding. The study was conducted across regional offices of the OfERR in cities of Chennai, Erode, Trichy, and Nella in Tamil Nadu, India. The study employed purposive sampling to interview participants from various refugee camps across Tamil Nadu. Sample size (n=15) comprised 60% male and 40% female, with an average age of 30, living among intergenerational family members. The participants expressed appreciation for the Indian Government’s support but voiced concerns about the Sri Lankan government’s lack of a concrete repatriation plan. Among the key challenges identified were the lack of infrastructure development, insufficient interventions to address intergenerational conflict within families, and concerns regarding resources for repatriation.

These findings align with the Conceptual Framework of Repatriation Success highlighting the importance of livelihood development, cultural awareness, social relationships, and equal citizenship for successful repatriation. Additionally, the PI and investigating team expanded on these findings by developing a policy framework for successful repatriation. They identified four key areas necessary for reintegration: livelihood development, language and cultural awareness, social relationships, and equal citizenship. Despite these policy recommendations, many refugees remained reluctant to return, citing the lack of infrastructure and support in Sri Lanka, which continued to contribute to psychological distress while living in India [18].

In summary, the qualitative study underscores the importance of addressing both the practical and psychological barriers to repatriation as they impact family dynamics. This provides a foundation for further exploration of how family relationships and intergenerational conflict influence broader psychosocial outcomes, linking directly to the focus of the family dynamics study, which examined the longitudinal associations between migration stressors, resource utilization, family dynamics, and health outcomes. Together, these two studies have contributed to understanding the complex interplay between repatriation readiness and family well-being among Sri Lankan Tamil refugees.

### 3.3. Study 3: Testing Family Dynamics Among Sri Lankan Tamil Refugees

**Figure 5:**
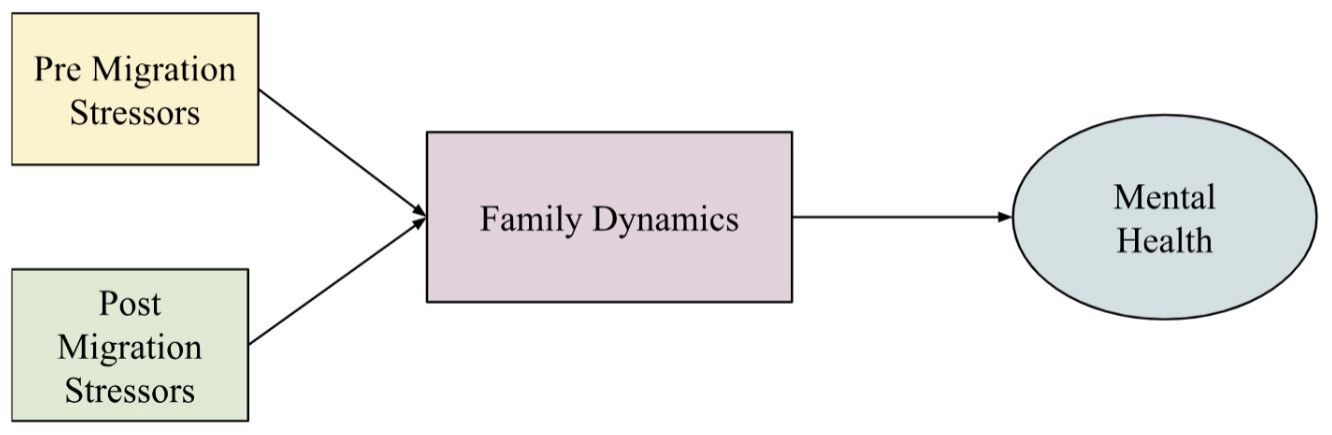
Longitudinal study of pre/post migration stressors on family dynamics and parental mental health.

The primary objective of the Family Dynamics (FD) study examined the longitudinal associations between migration stressors, resource utilization, family dynamics, and health outcomes among Sri Lankan Tamil refugees (2014–2016). The concept of family dynamics was operationalized to address the variables of communication and cohesion, parental responsiveness, and intergenerational conflict. The framework for this study is illustrated in Figure 5. Building upon the findings of the previous studies, this research aimed to identify areas for intervention to improve well-being, health outcomes, and social infrastructure within this population. By exploring the complex relationships between migration-related stressors, family cohesion, and psychological distress in both parents and adolescents, the FD study sought to inform culturally sensitive interventions that address family dynamics and resilience mechanisms among Tamil refugee families [35,36,37].

IRB approval was obtained (IRB #HM20000475; 11 March 2014) from the Virginia Commonwealth University and funded by National Institute of Health Fogarty International Early Career Award K01-TW 009648. Participants were selected through systematic random sampling, inviting every eighth household from a frame of 1500 families residing in the Trichy refugee camp. Eligibility criteria included parent-adolescent dyads living together in the camp and identifying as Sri Lankan Tamil refugees. Data collection involved 14 psychometrically validated measurements and included 120 parent-adolescent dyads. The data were collected in two phases over three years with Phase I spanning from June 2014 to April 2015, and Phase II from June 2016 to April 2017. Structured interviews were conducted in the local Tamil language by trained health workers in collaboration with OfERR, ensuring cultural appropriateness and community involvement. To maintain confidentiality and enhance data accuracy, parents and adolescents were interviewed separately.

The quantitative strand of this concurrent mixed-methods study utilized several standardized measures to assess refugee family dynamics, mental health, sleep quality, and pre/post-migration stressors. Qualitative data were analyzed using the thematic analysis method suggested in [38], incorporating both deductive and inductive approaches to capture family dynamics and cultural values. The PI and investigating team analyzed the impact of transmigration stress on mental well-being and family functioning by using paired *t*-tests to compare migration stressors, family functioning, and health outcomes between Phase I and Phase II of the study [35]. Additionally, they also developed a predictive model for refugee mental health using advanced machine learning/artificial intelligence (ML/AI) techniques, which were shown to outperform traditional linear regression methods [36]. The quantitative data analysis also included hierarchical linear regression and Extreme Gradient Boosting (XGBoost), a non-linear machine learning model, to examine the effects of child-specific variables, parental mental health, and family functioning on child depressive symptoms [37].

Worries about family (94.9%), poverty (91.6%), loneliness and boredom (71.4%), uncertainty about the future (87.5%), and lack of home ownership (89.8%) were the most commonly noted stressors within the sample. Transmigration stressors were significantly associated with psychological distress, somatic complaints, and depressive symptoms. The total transmigration stressor score was significantly correlated with various health outcomes and family functioning in expected directions: somatic complaints (r=0.38,p<0.001), depressive symptoms (r=0.47,p<0.001), hostility (r=0.47,p<0.001), and negatively with family functioning (r=−0.24,p=0.007). The study found a significant reduction in overall transmigration stressors between Phase I and Phase II ($M_{PhaseI}=54.49,SD=12.29;M_{PhaseII}=49.89,SD=10.16;p<0.01$), reflecting an improvement in family functioning over time. No significant changes were observed in sleep difficulties, somatic complaints, or hostility between phases.

Family dynamics, particularly family cohesion and parenting quality, were found to mediate the relationship between daily stressors and mental health outcomes. Hierarchical regression analysis demonstrated that family cohesion had a statistically significant role in mitigating mental health risks (β=−0.163,p<0.05). Refugees who utilized informal support from family and friends reported better family functioning, with resource utilization from Non-Governmental Organizations (NGOs) further improving outcomes [36]. The study found that 96% of participants reported using NGO resources such as OfERR, while 76.7% accessed informal family support. Refugee families who accessed formal NGO support reported lower depressive symptoms and better family functioning. Conversely, refugees who accessed support from family reported higher levels of family functioning (p=0.003) than those who did not use these resources.

Thematic analysis from in-depth interviews revealed key cultural values driving refugee family dynamics. Themes included patriarchal family structures, the importance of family honor, and the preservation of cultural identity. Parenting strategies, such as strict rules aimed at maintaining cultural values, often led to emotional strain for adolescents, reflecting tension between cultural preservation and adaptation to camp life. Additionally, family cohesion and collectivist values emerged as critical resilience factors, helping families cope with the challenges of displacement.

In summary, the FD study provided a comprehensive understanding of the complex effects of transmigration stressors on family dynamics and mental health in refugee families. The findings underscore the importance of culturally sensitive interventions that address family cohesion, resource utilization, and mental health support among Sri Lankan Tamil refugees. The development of a family functioning model highlights the role of family cohesion and mental health as mediators between migration stressors and child outcomes, providing a foundation for future interventions aimed at improving family well-being in refugee populations.

### 3.4. Study 4: A Grounded Theoretical Study of Parenting Processes and Intervention Development

The Parenting Processes and Intervention Development (PPID) Study, a qualitative design, focused on incorporating the cultural interpretations of the previous FD study results by community health workers (CHWs) within these communities. A qualitative exploratory design guided this PPID study, which was part of a larger concurrent mixed methods FD research project focusing on families from the Trichy refugee camp in Tamil Nadu, India (2017–2018). IRB approval was obtained (IRB #HM20000475; 11 March 2014) from the Virginia Commonwealth University and funded by National Institute of Health Fogarty International Early Career Award K01-TW 009648. Semi-structured interviews were designed specifically to explore the phenomenon of health workers in refugee camps. Purposive sampling was employed to recruit CHWs as participants of this PPID study. Participation was restricted to individuals in the camp who (1) worked as CHWs, and (2) who were Sri Lankan Tamil refugees. Two members of the research team conducted two focus groups (n=18). Recognizing that CHWs serve as both trusted insiders and frontline support providers, this study aimed to explore their unique perspective of family functioning, parenting practices, and resource utilization by the Tamil community within the refugee camp context.

Through in-depth discussions, the CHWs provided valuable insights into the cultural values, parenting practices, and familial dynamics prevalent among the refugees. Thematic analysis revealed “good” families are characterized by hard work, raising well-behaved children, helping those in need, and respecting elders. Maintaining family honor by not discussing internal problems with outsiders was deemed essential, reflecting a cultural norm that often led to reluctance to seek external support among family members. This reluctance hindered access to resources that could alleviate family conflicts and improve mental health outcomes.

Thematic analysis revealed authoritarian parenting styles were prevalent, with strict expectations of obedience and discipline from children. This approach frequently led to intergenerational conflicts, especially with adolescents born in the refugee camps who were more exposed to the host culture’s norms and values. The tension between preserving traditional Tamil cultural values and adapting to new environments contributed to family strain, echoing themes identified in the earlier studies. In addition, patriarchal structures remained influential within the refugee community, affecting gender roles and family responsibilities. While some shifts were noted, such as men contributing more to child-rearing, and domestic work largely remained the responsibility of women. The CHWs highlighted that these traditional roles could exacerbate family conflicts, particularly when compounded by the stresses of camp life and limited resources.

A significant challenge identified in the study was limited availability of trained counselors to address family conflicts effectively. CHWs themselves faced constraints due to a lack of training and resources, limiting their ability to support families adequately. They underscored the need for enhanced training in counseling, conflict resolution, and culturally sensitive intervention strategies. Building trust within the community was deemed crucial to encourage families to seek help without fearing the loss of family honor. Integrating these findings into the overarching research, PPID Study reinforced the central role of CHWs in bridging the gap between refugee families and support services. Their perspectives provided a deeper understanding of how transmigration stress impacts family functioning from both insider and service provider viewpoints. This PPID study complemented the previous studies by offering actionable insights into strengthening support systems within refugee camps, underscoring the importance of culturally sensitive interventions that address both individual and collective needs.

The findings from the PPID study contributed to the development of the intervention model for strengthening refugee family dynamics. This study, together with the findings from the PDMS study through FD study, provided empirical evidence on the multifaceted challenges faced by Sri Lankan Tamil refugee families. By integrating quantitative data on psychological distress, family functioning, and health outcomes with qualitative insights into cultural values, intergenerational conflicts, and resource utilization, the research illuminated the complex interplay between migration stressors, mental health and family dynamics and structure.

## 4. Discussion

**Figure 6:**
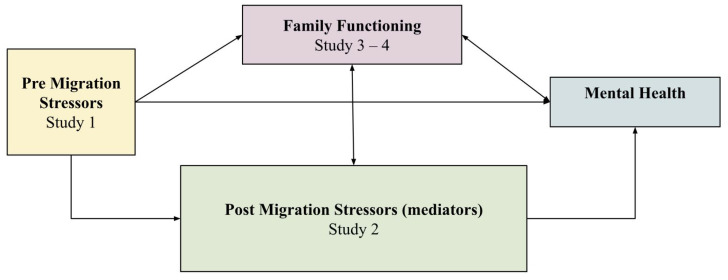
Conceptual Model of Refugee Family Dynamics.

The findings from the four studies provide critical insight into how pre- and post-migration stressors impact family dynamics and mental health among Sri Lankan Tamils in refugee camps (see Figure 6). In line with other ecological models [8,13,15,26], our data consistently show that pre-migration stressors, including traumatic war exposure, adverse psychosocial conditions, and material scarcity, emerged as a foundational source of mental health distress. Addressing the gap in the literature, this conceptual model centers the role of family functioning, situating it as a mediating factor of pre/post migration stress and mental health. While pre-migration stressors were found to be compounded by post migration stressors, each directly impacting mental health outcome, a key finding is the mediating capacity of family functioning on mental health.

Quantitative analysis indicates family demographics (e.g., larger families, lower parental education) negatively mediated mental health outcomes, while family functioning (communication and cohesion, parental responsiveness, and social support) emerged as a positive mediator to mental health. The reciprocal relationship between mental health and family dynamics was further evidenced in Study 4, where intervention by community health workers mediated the ongoing impact of resource scarcity present in the refugee camps. The evidence shows a correlation between resource scarcity and family functioning, with an increase in resources positively impacting families’ capacity for resilience. The collective evidence highlights family functioning as a key factor to understanding how resource constraints, intergenerational conflict, and broader community factors impact refugees’ psychological well-being.

Qualitative data across the studies identified three core family dynamic components significant to future interventions. First, parenting communication and cohesion shaped by the desire to preserve cultural continuity, were found to impose high levels of control, causing friction in the realm of adolescents adapting to host culture norms. Second, parental responsiveness is identified as a goal of the parents to keep the cultural identity, which manifests in parental voices silencing adolescents voices, impacting parental capacity to respond and establish open communication. Lastly, intergenerational conflict was identified due to both acculturation differences and the strain of long term displacement. These findings underscore the need for socio-emotional support to be given to families in addition to material resources and legal aid.

All four studies consistently demonstrate that resource utilization—both informal (family and friends) and formal (non-governmental organizations (NGOs) and community health workers) can enhance family functioning, reduce psychological distress, and improve overall well-being. Interventions aimed at enhancing the capacity of these support systems by developing and providing community collective interventions, increases access to resources such as counseling and health workers’ training can then amplify their impact on migration stressors, family dynamics and mental health outcomes. These findings contribute to existing research by emphasizing family functioning alongside material resource provision. Future research should focus on a holistic approach to effectively address the complex interplay of migratory stressors, family dynamics, and mental health among refugee populations experiencing protracted displacement.

**Figure 7:**
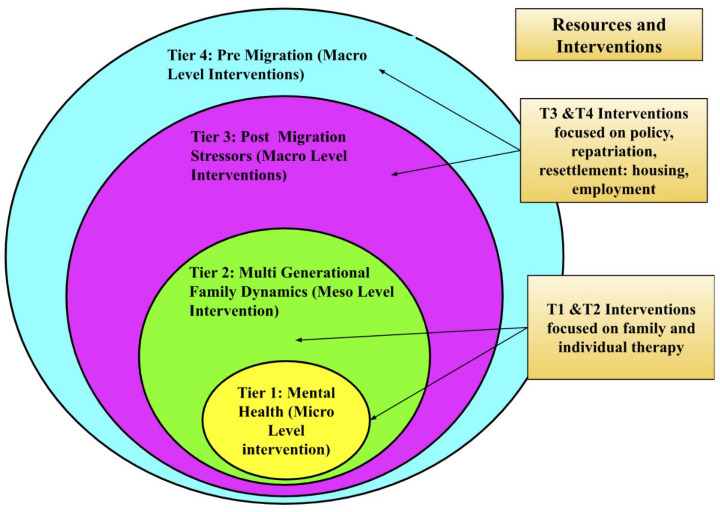
Framework for Refugee Health Intervention.

Based on the discussion above, we propose a variation of ecological models to address the four tiers identified in our research, illustrated by Figure 7. Future intervention models should integrate the four-tiered approach by addressing mental health at the **micro level**, multi-generational family dynamics at the **meso level**, and migration stressors and policies at the **macro level**. At the center, Tier 1 focuses on individual mental health intervention, recognizing that refugees often carry trauma from war, protracted displacement, and ongoing uncertainties that manifest as anxiety, depression, or post-traumatic stress. Tier 2 broadens this scope to multi-generational family dynamics, where intergenerational conflict and cultural preservation efforts may collide with adolescents’ adaptation to the host environment; interventions here should support family cohesion, open communication, and culturally responsive parenting strategies. Building outward, Tier 3 targets post-migration stressors—such as insecure legal status, limited housing, and employment barriers—through policy and resource-level solutions that stabilize daily life and reduce family conflict. At the outermost level, Tier 4 draws attention to pre-migration factors, including war exposure and forced displacement, by advocating for macro-level changes that address the underlying political and structural conditions leading to refugee crises. Taken together, all four tiers underscore the need for a holistic and sustained framework that coordinates mental health services, family-based interventions, social resources, and policy advocacy to effectively mitigate the long-term impact of forced displacement.

## Data Availability

The data sets used for the quantitative studies in this project can be found at https://github.com/indranil09/SLRefugees_MHPred and https://github.com/indranil09/Child-Mental-Health-Predictor (accessed on 22 January 2025).
